# Supplementary material for: Cross-species conservation in the regulation of parvalbumin by perineuronal nets
Source: Front Neural Circuits. 2023 Dec 19;17:1297643. doi: 10.3389/fncir.2023.1297643 (PMC10766385; doi:10.3389/fncir.2023.1297643)
Supplement: Supplementary file 1 [file Image_1.pdf]

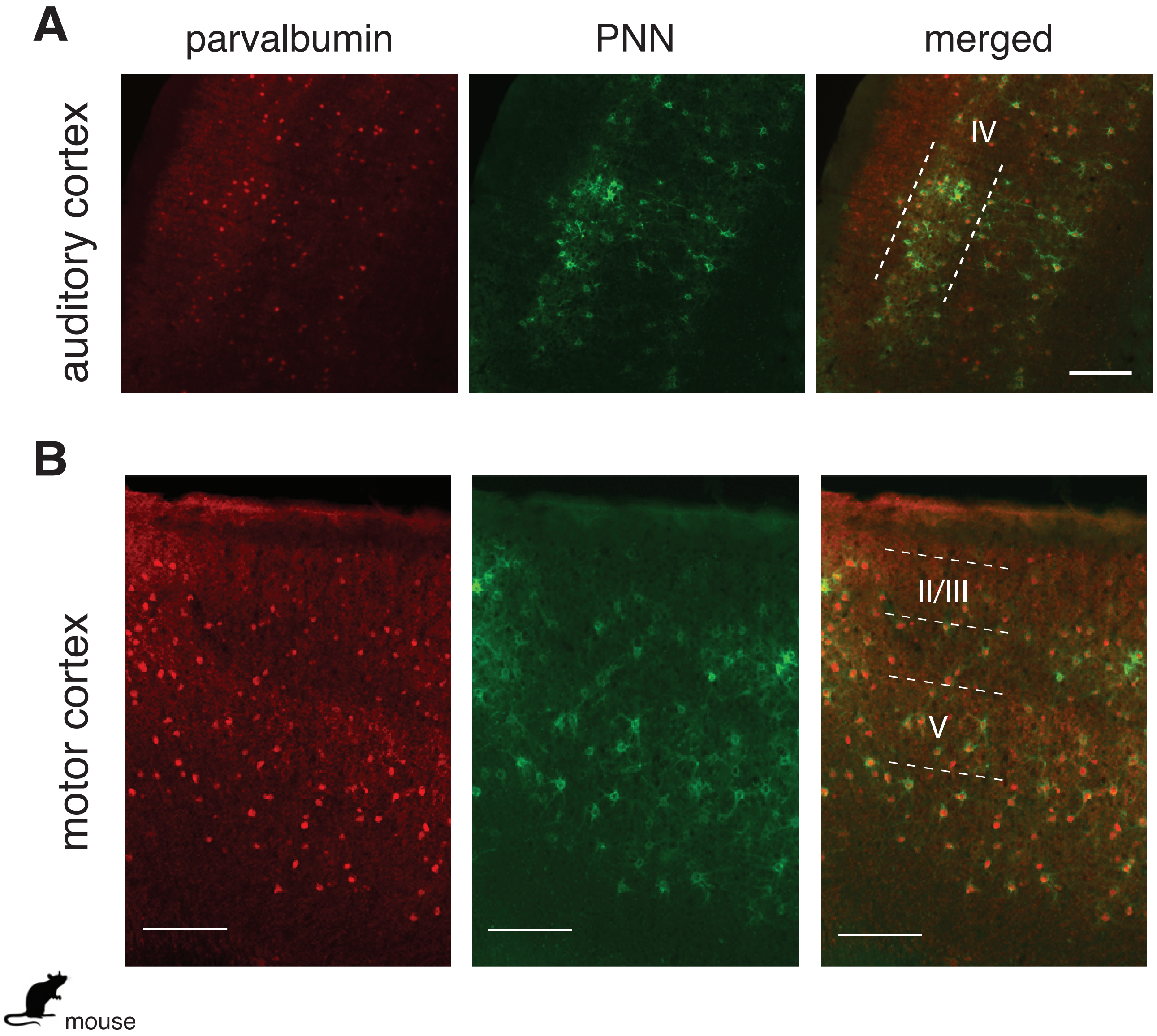

Supplementary Figure 1. Low magnification images of the auditory cortex (A) and motor cortex (B) of mice. We quantified PV intensities of PV neurons in layer IV of the auditory cortex and layers II/III and V of the motor cortex because these layers were most abundant for PV neurons and PNNs (e.g., Alpar et al., 2006; Fader et al., 2016; Lupori et al., 2023).
